# Statistical and Multivariate Evaluation of Olive Oil Degradation During Long-Term Storage

**DOI:** 10.3390/foods14234065

**Published:** 2025-11-27

**Authors:** Erislene S. Almeida, Danyel F. Silva, Natalia S. de Oliveira, Juliana S. Fernandes, Bruna C. S. Oliveira, Simone Monteiro, Fernanda V. Almeida, Jez W. B. Braga, Ana C. B. Dias

**Affiliations:** 1 Faculty of Technology, University of Brasilia—UnB, Brasilia 70910-900, Brazil; erislene.almeida@gmail.com (E.S.A.); simonems@unb.br (S.M.); 2 Institute of Chemistry, University of Goiás—UFG, Goiania 74690-900, Brazil; danyelfdsilva@gmail.com; 3 Institute of Chemistry, University of Brasilia—UnB, Brasilia 70910-900, Brazil; nataliaso2000@hotmail.com (N.S.d.O.); fernandaalmeida@unb.br (F.V.A.); jez@unb.br (J.W.B.B.); 4 Faculty of Health Sciences, University of Brasilia—UnB, Brasilia 70910-900, Brazil; juliana.sousa@unb.br

**Keywords:** chemometrics, oxidative degradation, olive oil stability, quality parameters, pharmaceutical excipients, principal component analysis (PCA)

## Abstract

Extra virgin olive oil (EVOO) is valued for its flavor and health benefits. However, its quality can decline during storage, reducing food quality and the effectiveness of therapeutic compounds when used as a pharmaceutical excipient. While the oxidative stability of extra virgin olive oil has been widely studied for food quality and shelf life, its crucial role as a pharmaceutical excipient and the impact of long-term degradation on the effectiveness and stability of active compounds remain largely unexplored. This study examined 14 commercial EVOO samples immediately after opening and after three years of storage. Standard methods were used to measure the peroxide value, p-anisidine value, acidity, antioxidant activity, and extinction coefficients. The data were analyzed with statistical and chemometric tools. Initially, all oils met international quality standards. After three years, most samples showed significant deterioration, especially a decline in antioxidant activity and an increase in K232 values. Statistical tests confirmed differences among samples, and pairwise comparisons indicated significant differences consistent with degradation between T0 and T1. Principal component analysis (PCA) identified three main patterns related to oxidation and antioxidant capacity, and clustering distinguished between stable and unstable samples. Overall, the stability of the studied EVOOs varied by brand, influenced by their natural composition and storage conditions. Multivariate analysis confirmed that antioxidant activity and extinction coefficients are key indicators of oxidative degradation. This finding highlights multivariate analysis as a valuable approach for monitoring the oxidative stability of oils and ensuring EVOO quality for both food and pharmaceutical application.

## 1. Introduction

Extra virgin olive oil (EVOO) is one of the most popular vegetable oils, valued for its appealing sensory qualities, health benefits, and perceived natural oxidative stability [1]. According to the International Olive Council (IOC), Extra Virgin Olive Oil (EVOO) is obtained exclusively from the fruit of the olive tree (*Olea europaea* L.) using only mechanical or physical methods. To keep its high quality, the process is carried out under controlled thermal conditions, and the oil undergoes no treatments besides washing, decanting, centrifugation, and filtration [2]. The lack of refining is essential for maintaining the oil’s distinctive sensory traits and, more importantly, its wide array of minor bioactive compounds, which would be mostly removed during refining.

EVOO’s composition is approximately 97% (*m*/*m*) of acylglycerols, with oleic acid being the primary esterified fatty acid. The remaining compounds consist of various minor non-glyceridic components such as aliphatic and triterpene alcohols, sterols, hydrocarbons, phenols, tocopherols, esters, pigments, and volatile compounds [3]. Among these, phenols and tocopherols are especially significant because of their high antioxidant activity, which is due to the presence of phenolic hydrogens that help slow down the oxidation of fatty acids by inhibiting free radical formation, or by interrupting their progression through creating more stable radicals via electron delocalization over conjugated aromatic ring systems [4].

The composition of these minor compounds in olive oils, as well as the overall fatty acid composition, can vary significantly in response to the olive tree to environmental parameters during cultivation and harvesting. Some studies present that trees cultivated in colder regions often yield oils with higher levels of antioxidants and monounsaturated fatty acids (oleic acid and the oleic/linoleic acid ratio) [5]. In contrast, those from warmer climates tend to be richer in saturated and polyunsaturated fatty acids (palmitic and linolenic acids) [5]. These correlations were observed in different types of genotypes, such as Arauco and Arbequina, highlighting the influence of climate on the biosynthesis of these compounds [6]. Beyond these antioxidants, free fatty acids are also naturally present in olive oil; their content is a key parameter usually expressed as acidity index (AI, g of free acidity expressed as oleic acid per 100 g of oil) used to distinguish between various grades, from extra virgin (with values up to 0.8) to lampante oil (with values exceeding 3.3) [2,7].

EVOO’s popularity and high price also make it vulnerable to adulteration with other oils. To ensure quality and prevent such practices, specific parameters must be met: acidity index (≤0.8), peroxide index (≤20.0 meqO_2_∙kg^−1^), and specific extinction coefficients in ultraviolet at 232 nm (≤2.50) and 268 nm (≤0.22) the latter two corresponding to the absorbance of a 1% (*w*/*v*) solution of oil in isooctane measured in a 1 cm path-length cell (AOCS Ch 5-91) [2]. Therefore, routine physical-chemical analyses are crucial for determining EVOO’s composition, detecting adulteration, and evaluating its degradation level, thus ensuring compliance with commercialization regulations.

Despite its inherent antioxidant activity, EVOO remains susceptible to degradation, which is usually triggered by poor storage conditions, exposure to light, and contact with atmospheric oxygen [8]. This results in the formation of primary oxidation products, like peroxides and hydroperoxides, and later, secondary oxidation products such as aldehydes, ketones, epoxides, alcohols, hydroxylated compounds, oligomers, and polymers [9]. Oxidation mainly occurs at the carbon next to the double bonds of fatty acids, although it can also affect the carbon next to the carboxylic group in saturated fatty acids [10].

Olive oil degradation, mainly through oxidative processes, causes undesirable changes in its flavor, color, and aroma. This deterioration can weaken the effectiveness and stability of active pharmaceutical ingredients (APIs) and biocompounds when EVOO is used as an excipient in medical formulations [11]. Such effects on quality and performance are especially important in pharmaceutical uses that involve sensitive compounds and vitamins, where product stability directly impacts therapeutic effectiveness. Because EVOO varies naturally and is prone to degradation over time, implementing strong methods for quality control and differentiating samples based on oxidative resistance is crucial.

Although the oxidative stability of extra virgin olive oil has been widely studied in terms of food quality, shelf life, and detecting adulteration, its crucial role as a pharmaceutical excipient remains underexplored. In pharmaceutical applications, the effects of degradation are compounded: oxidation processes not only affect sensory and nutritional qualities but, more critically, can also threaten the effectiveness and stability of solubilized medicines, particularly liposoluble active compounds and vitamins. While EVOO’s increasing use in lipidic drug formulations necessitates stringent quality control, the current literature remains deficient in comprehensive, long-term monitoring studies utilizing multivariate tools to systematically evaluate and predict EVOO’s continued suitability as an excipient under simulated usage conditions. Filling this gap is crucial for ensuring both therapeutic effectiveness and patient safety.

In this study, the oxidative degradation of 14 commercial EVOO at opening (T0) and after three years, used PCA/HCA to identify parameters distinguishing samples by oxidative stability for excipient suitability. Chemometric techniques, including Principal Component Analysis (PCA) and hierarchical clustering analysis, were used to identify key quality parameters that distinguish samples based on their oxidative stability.

## 2. Materials and Methods

### 2.1. EVOO Samples

Fourteen EVOO samples, representing different brands, countries of origin, bottling dates, and expiration dates (Table 1), were purchased randomly from different local supermarkets (Brasília, Brazil) within the same week, without intentional batch sampling, prioritizing the most recent bottling dates available.

### 2.2. Physical-Chemical Analyses

Analyses of antioxidant capacity and physicochemical quality parameters were performed at two time points: immediately after purchase and bottle opening (time zero, T0) and after a three-year storage period (time two, T1). During storage, the samples were kept in a closed, dark cabinet inside the research laboratory under climate conditions of 25 ± 3 °C. Quality parameters were determined following AOCS (American Oils Chemists’ Society) methodologies, including peroxide value (Cd 8b-90), aldehydes and other secondary oxidation derivatives by the p-anisidine method (Cd 18-90), free fatty acids (Ca 5a-40), and specific extinction coefficients (Ch 5-91). Antioxidant capacity was assessed using the DPPH method, which employs 2,2-diphenyl-1-picrylhydrazyl (Sigma-Aldrich, St. Louis, MO, USA) as a stable free radical A 9.35 × 10^−5^ mol L^−1^ DPPH solution was prepared by dissolving 36,869 mg in ethyl acetate to prepare a 100 mL solution. The procedure consisted of the addition of four drops of EVOO by using a 20 μL micropipette, in 4.0 mL of 9.35 × 10^−5^ mol L^−1^ DPPH solution. After mixing, the solution was maintained in a dark cabinet for 2 h. Then, the solution was transferred to a 1 cm quartz cuvette and analyzed in a UV-VIS spectrophotometer at 520 nm, which corresponds to the maximum absorption of DPPH free radical solution. The assay measures the capacity of antioxidant compounds in the oil to scavenge this radical, decreasing the initial absorbance.

The antioxidant activity (AA) of the oil is expressed as a percentage and is determined by the following Equation (1):

(1) $$AA=\frac{A_{DPPH}-A_{EVOO}}{A_{DPPH}}\times 100$$

where ADPPH = absorbance value of DPPH solution measured at 520 nm; AEVOO = absorbance value of DPPH with EVOO sample.

### 2.3. Statistical and Chemometric Analysis

Chemometric techniques, including Principal Component Analysis (PCA) and Hierarchical Clustering Analysis (HCA), were performed using Python version 3.13, with the scripts developed and executed in both Google Colab and the IDLE environment. They were applied to identify key quality parameters that distinguish samples based on their oxidative stability over a three-year storage period. The following section briefly describes the statistical and chemometric methods used to analyze the data and interpret the findings.

Preprocessing method for HCA and PCA: The mean values of the physicochemical parameters were autoscaled according to Equation (2) [12,13], since the parameters were expressed on different scales. In the present study, data were standardized using the traditional z-score transformation, which centers each variable by its mean and scales it by its standard deviation. Although [12,13] points out that the z-score may be less effective in some situations, this method was considered appropriate for our dataset because the variables present comparable scales and approximately normal distributions. Moreover, z-score normalization is widely adopted in multivariate analyses such as PCA and HCA, as it preserves the relative variance structure among variables without distorting their relationships. Preliminary tests using range-based scaling or using the Ward method did not lead to significant improvements in clustering or component separation, supporting the suitability of the z-score approach for this study. Without standardization, variables with higher magnitudes could dominate the Euclidean distance calculation, thereby distorting the formation of groups [12,13,14].

(2) $$z=\frac{M-\mu }{\sigma }$$

where z is the standardized value (dimensionless), M is the original measurement of a given variable, μ is the arithmetic mean of that variable across all samples, and σ is the corresponding standard deviation.

Hierarchical Clustering Analysis (HCA): the cluster analysis was performed on the autoscaled values of all physicochemical parameters using the Euclidean distance as similarity measure and the unweighted pair group method with arithmetic mean (UPGMA) as the linkage criterium [15].

Principal Component Analysis (PCA): PCA was performed in Python version 3.13 software to extract the most relevant parameters into a reduced number of variables. It is a chemometric model that explores interrelationships among numerous variables by compressing the systematic variation into the principal components, composed by the scores and loadings. This model allows the identification of hidden patterns present in the data through the transformation of experimental data tables into informative similarity-based graphical representations. [16].

Analysis of Variance (ANOVA): The comparison of physicochemical parameters among EVOO samples was performed using one-way ANOVA, since each parameter was evaluated independently. All factors were treated as fixed effects because the oils represent specific commercial samples rather than random draws from a broader population. Each sample contained five replicates (n = 5), as reported in the Results section for each parameter. Before running ANOVA, the assumptions of normality and homoscedasticity were tested using the Shapiro–Wilk and Levene tests, respectively. When the assumption of equal variances was violated, the Welch ANOVA was applied as a corrective approach. All ANOVA procedures were carried out using the Past version4.03 software [16].

Multiple comparisons (Tukey or Games–Howell): For datasets that met the ANOVA assumptions, post hoc comparisons were conducted using Tukey’s HSD test, which provides built-in control of Type I error. When Welch ANOVA was required due to heteroscedasticity, group comparisons were performed using the Games–Howell test, which does not assume equal variances or balanced sample sizes. All post hoc analyses were performed in th [16].

Student’s *t*-test: Pairwise comparisons between samples from the same origin evaluated at time zero and after three years were performed using independent Student’s *t*-tests. The assumptions of normality and variance equality were assessed using the Shapiro–Wilk and Levene tests. When unequal variances were detected, Welch’s *t*-test was applied instead of the standard *t*-test. A significance level of *p* < 0.05 was adopted. All *t*-tests were performed using Python version 3.13 [17], with scripts executed in Google Colab and the IDLE environment. No *p*-value adjustment was applied because each comparison involved only one pair of means per parameter per origin.

## 3. Results

### 3.1. Physical-Chemical Analysis

**Figure 1:**
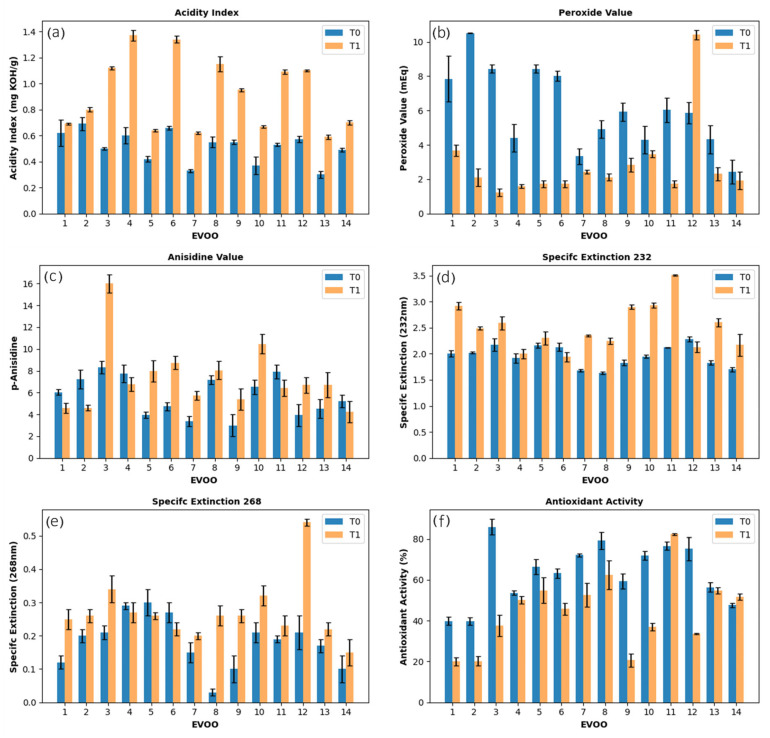
Evolution of physicochemical parameters of extra virgin olive oil (EVOO) samples analyzed (T0) immediately after opening the bottles and (T1) after three years of storage at room temperature, protected from light: (**a**) acidity index (AI), (**b**) peroxide value (PV), (**c**) p-anisidine value (p-AV), (**d**) specific extinction at 232 nm (K232), (**e**) specific extinction at 268 nm (K268), and (**f**) antioxidant activity (AA). Error bars: ±SD.

The EVOO samples were analyzed for physicochemical parameters at two time points: immediately after opening the bottles (T0) and after three years of storage (T1) in a closed, dark cabinet inside the research laboratory under climate conditions of 25 ± 3 °C. (Figure 1). In the initial analysis, all bottles were within their expiration date, whereas after three years, only two remained unexpired. Notably, even oils still within their expiration date exhibited signs of degradation, as discussed below.

Regarding the acidity index (AI), initial values were within the permitted limit for EVOOs (≤0.8 g of oleic acid 100 g^−1^ of oil), with five samples presenting AI below 0.5% (*w*/*w*) [18]. After three years, half of the samples remained within the ideal limit, while the other half showed an increase that exceeded this limit (Figure 1a), which can be attributed to exposure to oxygen after the bottles were opened, promoting oxidation and hydrolysis of triacylglycerols and the release of free fatty acids. This behavior was consistent with the reports of other studies [18]. Oxidation progressed during storage at room temperature, even when the material was protected from light, following an auto-oxidation pathway [19].

Peroxide value (PV) and p-anisidine value (p-AV) reflect the extent of oxidation at the early and later stages of lipid degradation, respectively [12,13]. PV quantifies hydroperoxides formed during the initial stages of oxidation, which can subsequently decompose into short-chain volatile compounds responsible for rancidity [14]. Oils with PV > 10 meq O_2_·kg^−1^, although still within IOC limits, may exhibit early signs of oxidative deterioration and are more prone to rancidity, whereas PV < 10 meq O_2_·kg^−1^ indicates good oxidative stability and minimal formation of primary oxidation products. Most samples initially exhibited PV below this threshold, reflecting good stability. Over time, PV decreased at T1, consistent with hydroperoxide decomposition and formation of secondary products, indicating progression through the classical phases of oxidation: initiation (slow hydroperoxide formation), propagation (rapid hydroperoxide accumulation), and termination (hydroperoxide decomposition surpassing formation) (Figure 1b) [20,21]. Concurrently, p-AV, which measures secondary oxidation products such as aldehydes and ketones, increased in most samples as hydroperoxides decomposed. However, a few samples showed decreases in p-AV, which may be explained by reactions with other compounds (Figure 1c) [22]. Together, these results indicate that the EVOOs had largely entered the termination phase of lipid oxidation by the end of the storage period.

Specific extinction (K), measured at 232 nm (K232) and 268 nm (K268), provides additional insight into oxidative stability, reflecting primary (conjugated dienes) and secondary (conjugated trienes) oxidation, respectively [23]. Initially, all samples were within the recommended limits (K232 ≤ 2.5; K268 ≤ 0.22), indicating low levels of oxidation. After three years, six samples exceeded the K268 limit, while the number of samples above the K232 threshold doubled, although the overall variation was moderate (Figure 1d,e). These findings suggest that, although the oils were initially in an early stage of oxidation, both primary and secondary oxidative products accumulated over time, consistent with the trends observed in PV and p-AV measurements.

Finally, the progressive chemical changes were observed in antioxidant activity (AA). Initially, eleven samples exhibited AA above 50%, with six exceeding 70%. Then, after three years, only seven samples retained AA above 50%, and just one remained above 70% (Figure 1f). This decline reflects the degradation of phenolic compounds over time. Hydroxytyrosol, a major phenolic in olive oil, is particularly prone to degradation under typical storage conditions, whereas tyrosol is more stable [21]. This difference is attributed to the chemical nature of hydroxytyrosol, which contains a catechol group that undergoes spontaneous auto-oxidation in the presence of oxygen and is also a more reactive substrate for polyphenol oxidase. In contrast, tyrosol is a monophenol, and therefore less susceptible to oxidative degradation [24]. Consequently, oils with higher initial hydroxytyrosol content experienced a greater reduction in antioxidant activity. Minor variations in antioxidant activity may stem from analytical variability, as factors such as CO_2_ uptake or free fatty acids can affect DPPH results [21]. The increase in acidity index (AI) in all EVOO samples confirms ongoing degradation, supporting this interpretation.

Overall, the combined evaluation of PV, p-AV, SE, and antioxidant activity suggests that oxidative degradation progressed over the storage period, as evidenced by the differences between the initial and final states. While the oils were initially in an early oxidation state, both primary and secondary oxidation products accumulated over time, accompanied by a decline in phenolic-driven antioxidant activity. These variations among samples highlight differences in their oxidative stability, providing a strong rationale for the subsequent chemometric analysis, which aims to identify key quality parameters and patterns that discriminate samples based on their resistance to oxidation.

The interaction among parameters proved complex. Although phenolic compounds act as antioxidants and delay peroxide formation, antioxidant activity (AA) and peroxide value (PV) did not display consistent patterns among samples. Variability across samples suggests that oxidative stability is governed by multiple interacting factors, such as fatty acid composition, metals, pigments, oxygen, and storage conditions, rather than a simple linear relationship [25,26]. This highlights the importance of proper storage to preserve phenolic compounds, which are essential for maintaining the functional and sensory quality of EVOOs.

### 3.2. Pattern Recognition Using HCA and PCA

EVOO samples were characterized through well-established physicochemical analyses, all of which demonstrated progressive oxidation during storage. However, the univariate analysis of these parameters did not allow a clear conclusion about the separation between the two storage periods, considering simultaneous interpretation of six parameters or revealing the correlations between the quality parameters. Therefore, a multivariate approach based on HCA and PCA was applied to integrate the data and reveal overall compositional patterns.

**Figure 2:**
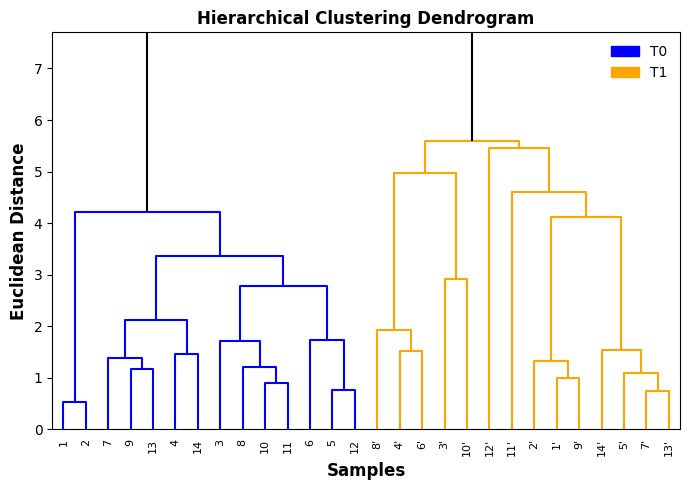
Hierarchical clustering (Euclidean distance, UPGMA) of extra virgin olive oil (EVOO) samples analyzed immediately after opening the bottles and after three years of storage at room temperature, protected from light. Samples from the second time point are denoted with an apostrophe (′) and dotted line corresponds to XXX.

Figure 2 shows the dendrogram obtained from the Hierarchical Cluster Analysis (HCA), where the formation of two main clusters can be observed at a Euclidean distance of approximately 5.7. This separation highlights the presence of clear differences between the two sets of samples, corresponding to the freshly opened oils (without an apostrophe) and the oils stored for three years (identified with an apostrophe). The fact that all samples with an apostrophe are concentrated in the same group, distinct from the original samples, demonstrates that storage time had a significant impact on the chemical profile of the oils, consistently altering their properties over the study period.

The internal structure of each cluster shows that, although the oils within the same period present general similarities, there are still individual variations among them. This may be related to differences in the original composition of the oils, such as fatty acid proportions, levels of natural antioxidant compounds, and residual water content, as well as possible variations in storage conditions before the acquisition of the olive oils [20,21,24]. Even within a time-homogeneous group, the presence of subclusters indicates that factors such as packaging type, light exposure, and minor compounds influenced by product origin may also have influenced the observed chemical changes.

Within the first main cluster, which contains the freshly opened samples, the linkage distances are shorter, suggesting greater chemical similarity among them. This indicates that the oils were relatively homogeneous before storage, presenting a similar behavior, which agrees with the similar values obtained for most quality parameters (p-anisidine, acidity, K232, and K268 in Figure 1). In contrast, the second main cluster, composed of the samples stored for three years, displays longer branch lengths, revealing greater internal variability. This can be explained by the fact that oxidative processes progress at different rates depending on the individual composition and storage history of each sample.

The clear separation between the two main clusters also indicates that the combination of all evaluated parameters provided a consistent discrimination between fresh and aged oils. The Euclidean distance used in the analysis reflects the magnitude of the overall chemical change, and the large gap between the clusters shows that the transformation was not restricted to a single variable, but rather a cumulative effect involving multiple degradation indicators. This reinforces the robustness of the multivariate approach for distinguishing oils at different stages of oxidative deterioration.

PCA was applied to reduce data dimensionality and correlate the patterns observed in the samples with the variability among the physicochemical parameters. The first three principal components (PC1, PC2, and PC3) explained 75.57% of the total variance (Table 2), which represents most of the systematic variation in the dataset. In addition, inspection of the scores of the last three components (PC4–PC6) did not reveal any systematic pattern.

**Figure 3:**
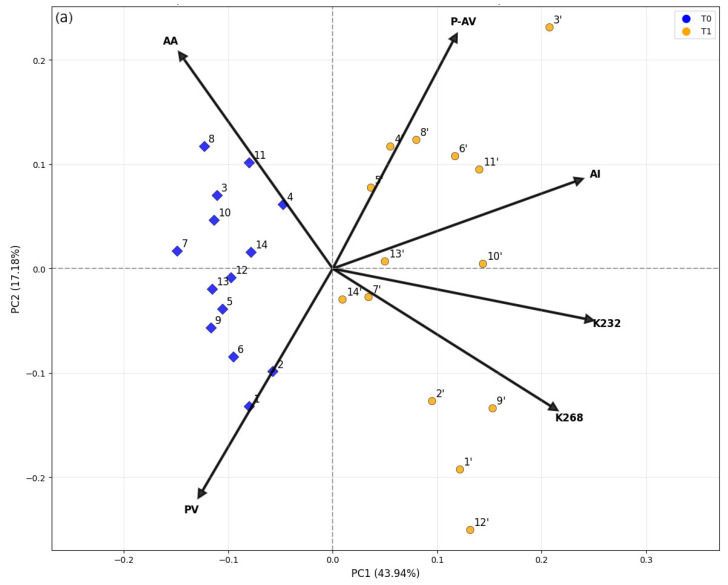

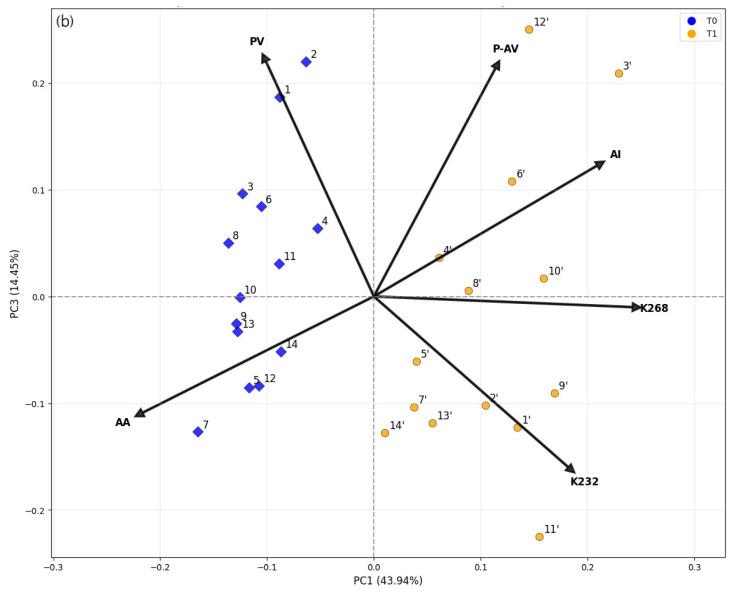
Score plots from Principal Component Analysis (PCA) of extra virgin olive oil (EVOO) samples: (**a**) PC1 vs. PC2 and (**b**) PC1 vs. PC3. Samples were analyzed immediately after bottle opening (blue symbols) and after three years of storage at room temperature, protected from light (yellow symbols). Arrows indicate loading values, with labels for both the loadings and the samples.

The biplot presented in Figure 3a shows that all variables exhibited relevant loadings values (>0.1) on PC1, which accounted for 44.0% of the total variance. Antioxidant activity (AA) and peroxide value (PV) were correlated and contributed to negative PC1 scores, whereas p-anisidine value (p-AV), acidity index (AI), extinction coefficient at 232 nm (K232), and 268 nm (K268) were also correlated but contributed to positive scores. This component was primarily responsible for the clear separation between samples analyzed immediately after bottle opening (T0, blue) and those analyzed after three years of storage (T1, yellow), with all T0 samples exhibiting negative PC1 scores and all T1 samples showing positive ones. T1 showed advanced oxidation signatures (higher K232/K268, higher p-AV) with reduced PV due to peroxide breakdown. Conversely, freshly opened samples clustered at the negative score values of PC1, reflecting lower degradation and higher antioxidant activity (AA). The changes in the peroxide value (PV) and extinction coefficient at 232 nm (K232) are both related to primary oxidation through hydroperoxide and conjugated diene formation [22,27], whereas the extinction coefficient at 268 nm (K268) reflects the progression toward secondary oxidation [26]. PC2 (17.2%) captured additional variation related to hydrolytic and secondary oxidation: positive scores corresponded to higher p-AV and AI values, while negative scores were associated with PV and K268, indicating samples with a stronger contribution of oxidative products (primary and secondary) rather than hydrolytic changes [20]. Antioxidant activity (AA) also contributed positively to PC2, suggesting that some oxidized samples retained measurable antioxidant capacity. This residual activity likely results from synergistic interactions between phenolic and non-phenolic antioxidants, as described by Bendini et al. [28]. One possible explanation is that phenols with ortho-dihydroxyl structures, such as tyrosol derivatives, can regenerate oxidized α-tocopherol forms, thereby prolonging antioxidant effectiveness even under moderate oxidative stress.

PC3 (14.4% of the variance) accounted for additional variation associated with intermediate oxidative stages. Positive loadings of PV and p-AV, combined with a negative loading of K232, indicate that samples with high PC3 scores contained both primary and secondary oxidation products, whereas low PC3 scores corresponded to oils dominated by conjugated-diene structures formed at early stages of lipid peroxidation. This component thus differentiates oils undergoing simultaneous accumulation and transformation of oxidation products, consistent with advanced auto-oxidation pathways [20].

In addition to the clear separation between samples analyzed immediately after bottle opening and those stored for three years, specific sample behaviors were evident. Among the aged oils, samples 1′, 2′, 9′, and 12′ showed high PC1 and negative PC2 scores, consistent with a stronger contribution of primary/advanced oxidation markers (PV and K268). In contrast, samples 4′, 5′, 6′, 8′, 11′, and 3′ stood out with positive PC2 scores, reflecting pronounced secondary oxidation and higher acidity (p-AV and AI). Notably, 3′ and 11′ combined high PC1 with positive PC2, indicating simultaneous increases in K268 and AI; 12′, however, primarily aligned with K268/PV rather than AI. Samples analyzed immediately after opening displayed lower dispersion, reflecting greater compositional similarity at this stage. Overall, PCA indicated that oxidative degradation (primary to secondary) and antioxidant preservation were the main drivers of sample separation. The combined interpretation of the 2D biplots and 3D PCA confirmed that storage for three years significantly altered the oils’ chemical profiles, although with heterogeneous effects among samples, likely due to intrinsic compositional differences and pre-acquisition storage conditions.

The application of Principal Component Analysis (PCA) to reduce dimensionality and identify the main sources of variability in olive oil samples is a widely used and validated approach in the recent literature. In this study, the first principal component explained most of the total variance, mainly reflecting the strong correlation between oxidation indices and antioxidant activity, thus providing a meaningful summary of the oxidative status of the samples. Mancebo-Campos et al. [29] demonstrated that variables related to oxidation and antioxidant constituents account for a large portion of the variability in olive oil shelf-life models, corroborating the justification for using PC1–PC3 to summarize the system’s behavior [29,30].

The finding that PC1 is mainly associated with primary oxidation markers such as PV and K232, with an additional contribution from K268 related to secondary oxidation, aligns with results from EVOO storage studies that identified exactly these variables as those with the highest loadings in the components that discriminate samples by time and degradation level. Caipo et al. [31] in a study on EVOO from the Arbequina cultivar under different storage conditions, showed that PV and spectral coefficients at 232 and 268 nm clearly evolve over time and contribute to the separation of samples [31].

The association of PC2 with acidity and p-anisidine, representing hydrolytic degradation and secondary oxidation, is equally supported in the literature. Studies modeling oxidation progression in olive oils have found that secondary oxidation and acidity indicators often load on distinct components or appear as relevant vectors in biplots, reflecting degradation processes complementary to those detected by PV and K232. These observations support the interpretation that PC2 captures oxidation aspects that are not identical to those of PC1 [29,32].

The emergence of a component dominated by antioxidant activity, independent of direct oxidation markers, has also been documented. Lobo-Prieto et al. [33] demonstrated that phenolic compounds and pigments can load separately on principal components and act as protective factors that modulate the rate and extent of oxidation. This behavior adequately explains why one PC (negative PC1 in this study) may primarily reflect antioxidant capacity and why samples with negative scores in this component show greater oxidation resistance.

The clear separation observed between freshly opened and stored samples in the PCA biplots is consistent with temporal monitoring studies that show score shifts as storage time increases or storage conditions change. Monthly monitoring or accelerated storage experiments record score migration patterns in the direction of oxidation and phenol loss vectors, very similar to the shift toward more positive PC1 and PC2 values [29,31].

Finally, the heterogeneity among samples and the influence of intrinsic composition or cultivar on degradation trajectories, observed in paired samples that either maintain their similarity or shift significantly after degradation, corroborates recent investigations showing genotype-dependent responses and dependence on initial phenolic composition. Vendrell Calatayud et al. [34] and other authors demonstrated that cultivar differences and initial levels of phenols and pigments strongly influence evolution during storage, explaining the inter-sample variability documented in the dataset [33,34].

### 3.3. Statistical Comparison of EVOO Quality Parameters

Analysis of variance (ANOVA) was used to determine whether the differences among olive oil samples were statistically significant for each parameter and across the two analysis periods. ANOVA confirmed significant differences for all parameters, with F values exceeding the critical value (1.8993, degrees of freedom equal to 13 and 56) and *p* < 0.05. Since no outliers were excluded in the analysis, the same critical value was used for all ANOVA significant tests. Based on these results, Tukey’s test was employed to identify specific pairwise differences. Out of 91 possible comparisons, more than half were significantly different in the first period for all parameters except K268. In the second period, antioxidant activity (AA), acidity value (AV), and K232 also showed significant differences in more than half of the sample pairs (Table 3).

Table 4 presents the comparison of mean values for each olive oil sample and parameter, obtained immediately after bottle opening (n) and after three years of storage (n′). Statistically significant differences (*p* ≤ 0.05), identified by the *t*-test for comparison of two means, are shown in bold. Antioxidant activity (AA) showed significant differences in 13 of the 14 pairs, consistent with oxidative degradation or loss of phenolic compounds over time. This finding is supported by previous studies, which report that phenolic compounds are particularly susceptible to degradation during storage, especially under thermal stress, resulting in a marked reduction in their concentration [21]. In addition to abiotic factors, this degradation is also driven by the action of polyphenol oxidase (PPO) naturally present in olive oil, which catalyzes the oxidation of catecholic phenols such as hydroxytyrosol [24]. The acidity value (AV) was less sensitive to temporal variation, with only 7 of the 14 samples showing significant differences, suggesting greater stability against triacylglycerol hydrolysis. The peroxide value (PV) showed significant differences in 12 of 14 pairs, with reductions in 11 of them. The exception was sample 12′, which exhibited a significant increase, suggesting decomposition of hydroperoxides into secondary products. The extinction coefficients K232 and K268 and the p-anisidine value (p-AV) were also frequently affected: K232 showed significant differences in 11 of 14 pairs, with 9 increases; K268 in 10 of 14 pairs, all with increases; and p-AV in 11 of 14 pairs, of which 8 showed increases.

This set of results, characterized by the predominant decrease in AA, consistent increase in AV, elevation of extinction coefficients and p-AV, and in many cases a reduction in PV, is consistent with the progression of oxidation during storage. This process involves the consumption of antioxidants and phenolic compounds, as well as the transformation of hydroperoxides into secondary products such as aldehydes and ketones, which explain the increases in K268 and p-AV, while PV may decrease due to peroxide decomposition. The exceptions observed in samples 11′ and 14′, which showed an increase in antioxidant activity (AA), and in sample 12′, which exhibited an increase in peroxide value (PV), indicate that not all samples followed the predominant trend between T0 and T1. Such deviations are expected given the heterogeneous nature of commercial EVOOs, potential matrix-related influences, and normal analytical variability. These exceptions do not alter the overall interpretation of the storage effects observed in the dataset. These findings are consistent with previous reports describing the susceptibility of phenolic compounds to degradation during storage [21].

Meanwhile, noticeable changes in both the p-anisidine value (p-AV) and peroxide value (PV) across most samples further confirmed the presence of ongoing oxidative processes. These findings align with those reported by [27], although that study evaluated short-term storage (3 months), which is substantially shorter than the 3 years assessed here. Their findings support the general notion that free fatty acid formation is less time-dependent than oxidative reactions during storage, while hydrolysis is more strongly influenced by factors such as temperature and water content. Oxidation, in contrast, proceeds via chain reactions that can occur even at ambient temperatures and low oxygen levels [27].

## 4. Conclusions

This study evaluated the oxidative stability of 14 commercial extra virgin olive oils intended for potential pharmaceutical use as excipients. EVOO was analyzed at two time points: immediately after opening and after three years of storage. Physical-chemical parameters were evaluated using AOCS standard methods, complemented by statistical and chemometric techniques (ANOVA, Tukey test, Student’s *t*-test, PCA, and hierarchical clustering). The results showed a progressive decline in quality over time, with antioxidant activity experiencing the most significant reduction. Extinction coefficients also reflected notable oxidative changes, while acidity remained comparatively more stable. PCA and dendrogram analyses confirmed that oxidation- and acidity-related parameters were key in distinguishing samples and monitoring degradation pathways. Although most oils deteriorated, some demonstrated greater resistance, indicating that intrinsic composition strongly influences stability. Overall, these findings highlight the importance of continuous monitoring of oxidative markers and the application of multivariate statistical tools to predict shelf life. While such changes may be acceptable from a food quality perspective, since the oils remained at relatively low oxidation levels, they can be critical when EVOO is used as a pharmaceutical carrier, where even minor oxidative alterations may promote the oxidation of incorporated drugs and compromise their biological functionality. Therefore, systematic monitoring of oxidation progression is essential to ensure the quality and efficacy of olive oil-based excipients in pharmaceutical and nutraceutical formulations.

## Figures and Tables

**Table 1:** Characteristics of Commercial Extra Virgin Olive Oil Samples: Origin, Packaging, Bottling, and Expiration Dates (mm/dd/yyyy).

Sample	Origin	Bottled	Expiration Date
**1 **	Argentina	02/03/2022	02/03/2024
**2**	Argentina	07/01/2021	07/01/2023
**3**	Tunisia	01/14/2022	01/13/2024
**4**	Portugal	01/18/2022	01/18/2024
**5**	Portugal	01/21/2022	07/31/2023
**6**	Portugal	01/06/2022	01/06/2024
**7**	Chile	03/26/2022	03/26/2024
**8**	Spain	02/01/2022	08/01/2025
**9**	Spain	01/24/2022	09/24/2023
**10**	Spain	07/31/2021	07/31/2023
**11**	Tunisia	01/25/2022	01/24/2024
**12**	Tunisia	02/07/2022	02/06/2024
**13**	Portugal	05/24/2022	01/31/2024
**14**	Chile	03/11/2022	03/11/2025

**Table 2:** Percentage of variance explained by the principal components extracted from PCA applied to the physicochemical parameters of extra virgin olive oil (EVOO) samples.

	Explained Variance (%)	Accumulated Variance (%)
PC 1	43.95	43.95
PC 2	17.21	61.16
PC 3	14.41	75.57
PC 4	11.16	86.72
PC 5	8.63	95.35
PC 6	4.65	100.00

**Table 3:** Results of Tukey’s test applied to mean values of physicochemical parameters in extra virgin olive oil (EVOO) samples.

Parameters	Number of Pairs of Samples Significantly Different from Each Other	
	Immediately After Opening	After 3 Years Opening
AA	77	72
p-AV	53	36
PV	53	40
AV	54	73
K232	63	62
K268	48	31

**Table 4:** Mean values (±SD) of physicochemical parameters in extra virgin olive oil (EVOO) samples analyzed immediately after opening the bottles and after three years of storage at room temperature, protected from light. Samples from the second time point are denoted with an apostrophe (′). Values in bold indicate significant differences (*p* ≤ 0.05), while the remaining values indicate no significant difference (*p* > 0.05).

Samples	AA(%)	AI (%)	p-AnV	PV (meqO_2_∙kg^−1^)	K 232	K 268
**1 **	**40.0 ± 2.0**	0.62 ± 0.10	**6.0 ± 0.3**	**7.9 ± 1.3**	**2.00 ± 0.06**	**0.12 ± 0.02**
**1′**	**20.0 ± 1.9**	0.69 ± 0.01	**4.6 ± 0.5**	**3.7 ± 0.3**	**2.9 ± 0.1**	**0.25 ± 0.03**
**2**	**39.7 ± 1.9**	**0.69 ± 0.05**	**7.2 ± 0.9**	**10.5 ± 0.8**	**2.02 ± 0.02**	**0.20 ± 0.02**
**2′**	**20.3 ± 2.3**	**0.80 ± 0.02**	**4.6 ± 0.3**	**2.1 ± 0.5**	**2.49 ± 0.03**	**0.26 ± 0.02**
**3**	**85.8 ± 1.0**	**0.50 ± 0.01**	**8.3 ± 0.6**	**8.4 ± 0.2**	**2.17 ± 0.12**	**0.20 ± 0.02**
**3′**	**35.0 ± 3.7**	**1.11 ± 0.01**	**16.0 ± 0.8**	**1.2 ± 0.2**	**2.6 ± 0.1**	**0.34 ± 0.04**
**4**	**53.6 ± 1.1**	**0.59 ± 0.06**	7.6 ± 0.8	**4.4 ± 0.8**	1.9 ± 0.1	0.26 ± 0.05
**4′**	**50.2 ± 1.9**	**1.37 ± 0.04**	6.8 ± 0.6	**1.6 ± 0.1**	2.00 ± 0.09	0.27 ± 0.03
**5**	**63.7 ± 1.3**	**0.41 ± 0.02**	**4.0 ± 0.3**	**8.4 ± 0.2**	2.16 ± 0.05	0.30 ± 0.04
**5′**	**58.4 ± 1.8**	**0.64 ± 0.01**	**8.0 ± 1.0**	**1.7 ± 0.2**	2.30 ± 0.13	0.26 ± 0.01
**6**	**62.4 ± 1.6**	**0.66 ± 0.01**	**4.7 ± 0.4**	**8.0 ± 0.3**	**2.13 ± 0.08**	0.27 ± 0.03
**6′**	**45.8 ± 2.9**	**1.38 ± 0.03**	**8.8 ± 0.6**	**1.7 ± 0.2**	**1.9 ± 0.1**	0.22 ± 0.02
**7**	**72.1 ± 0.8**	**0.33 ± 0.01**	**3.4 ± 0.5**	**3.3 ± 0.5**	**1.68 ± 0.03**	**0.15 ± 0.03**
**7′**	**49.4 ± 3.1**	**0.62 ± 0.01**	**5.7 ± 0.4**	**2.4 ± 0.1**	**2.35 ± 0.02**	**0.20 ± 0.01**
**8**	**80.8 ± 1.9**	**0.54 ± 0.04**	7.2 ± 0.4	**4.9 ± 0.5**	**1.63 ± 0.02**	**0.03 ± 0.01**
**8′**	**67.8 ± 6.9**	**1.14 ± 0.06**	8.1 ± 0.8	**2.1 ± 0.2**	**2.25 ± 0.06**	**0.26 ± 0.03**
**9**	**60.8 ± 1.6**	**0.55 ± 0.02**	**3.0 ± 1.0**	**5.9 ± 0.5**	**1.83 ± 0.05**	**0.10 ± 0.04**
**9′**	**20.6 ± 3.1**	**0.95 ± 0.01**	**5.4 ± 1.0**	**2.8 ± 0.4**	**2.90 ± 0.04**	**0.26 ± 0.02**
**10**	**72.0 ± 2.1**	**0.37 ± 0.07**	**6.5 ± 0.7**	4.3 ± 0.8	**1.95 ± 0.03**	**0.21 ± 0.03**
**10′**	**37.1 ± 1.8**	**0.67 ± 0.01**	**10.5 ± 0.9**	3.5 ± 0.2	**2.93 ± 0.05**	**0.32 ± 0.03**
**11**	**76.6 ± 2.0**	**0.53 ± 0.01**	**7.9 ± 0.6**	**6.0 ± 0.7**	**2.12 ± 0.01**	**0.19 ± 0.01**
**11′**	**82.2 ± 0.4**	**1.09 ± 0.02**	**6.4 ± 0.8**	**1.7 ± 0.2**	**3.51 ± 0.01**	**0.23 ± 0.03**
**12**	**75.2 ± 5.6**	**0.57 ± 0.02**	**3.9 ± 1.0**	**5.9 ± 0.6**	**2.28 ± 0.05**	**0.21 ± 0.05**
**12′**	**33.7 ± 0.4**	**1.11 ± 0.01**	**6.7 ± 0.7**	**10.4 ± 0.3**	**2.14 ± 0.10**	**0.54 ± 0.01**
**13**	56.4 ± 2.4	**0.30 ± 0.03**	**4.5 ± 0.9**	**4.3 ± 0.8**	**1.83 ± 0.04**	**0.17 ± 0.02**
**13′**	54.8 ± 1.4	**0.58 ± 0.02**	**6.7 ± 1.2**	**2.3 ± 0.4**	**2.60 ± 0.08**	**0.22 ± 0.02**
**14**	**47.6 ± 1.0**	**0.49 ± 0.01**	5.2 ± 0.6	2.4 ± 0.7	1.70 ± 0.04	0.10 ± 0.04
**14′**	**51.7 ± 1.5**	**0.70 ± 0.02**	4.3 ± 1.0	1.9 ± 0.5	2.13 ± 0.21	0.15 ± 0.04

## Data Availability

The original contributions presented in this study are included in the article. Further inquiries can be directed to the corresponding author.
